# Ultrasound-Targeted Microbubble Destruction-Mediated Inhibition of Livin Expression Accelerates Ovarian Cancer Cell Apoptosis

**DOI:** 10.1155/2021/7624346

**Published:** 2021-12-09

**Authors:** Xiaolin Xu, Shuqin Yu, Xiaoyuan Liu, Ying Feng

**Affiliations:** ^1^Department of Obstetrics and Gynecology, Shangrao Municipal Hospital, Shangrao, Jiangxi 334000, China; ^2^Department of Obstetrics and Gynecology, The Second Affiliated Hospital of Nanchang University, Nanchang, Jiangxi 330006, China

## Abstract

**Objective:**

Ultrasound-targeted microbubble destruction (UTMD) technique has recently been developed as a nonviral delivery of gene therapy. This study aimed at investigating the survival and apoptosis of ovarian cancer cell line OVCA-433 by inhibiting Livin expression through ultrasound-targeted microbubble destruction.

**Methods:**

We synthesized a targeted microbubble agent for UTMD-mediated shRNA against Livin gene in human ovarian cancer OVCA-433 cells. Lipid microbubbles were conjugated with a luteinizing hormone-releasing hormone analog (LHRHa) by an avidin-biotin linkage to target the ovarian cancer OVCA-433 cells expressing LHRH receptors. The microbubbles were mixed with the recombinant plasmid harboring shRNA-Livin. shRNA-Livin was transfected into OVCA-433 cells upon exposure to 1 MHz pulsed ultrasound beam (0.5 W/cm^2^) for 8 s. Cell survival was measured by the MTT assay, cell apoptosis by flow cytometry using annexin V/PI double staining, and cell ultrastructure by using the transmission electron microscope. The mRNA and protein expression levels of caspase-3 and caspase-8 were detected by RT-qPCR and western blotting.

**Results:**

UTMD-mediated delivery of shRNA-Livin remarkably reduced the survival of OVCA-433 cells but promoted the apoptosis compared with shRNA-Livin alone, shRNA-Livin plus nontargeted microbubbles, and shRNA-Livin plus LHRHa-conjugated microbubbles containing shRNA-Livin with or without exposure to ultrasound pulses. It was also found that UTMD-mediated delivery of shRNA-Livin notably declined the mRNA and protein expression levels of caspase-3 and caspase-8 in OVCA-433 cells compared with shRNA-Livin alone, shRNA-Livin plus nontargeted microbubbles, and shRNA-Livin plus LHRHa-conjugated microbubbles containing shRNA-Livin with or without exposure to ultrasound pulses.

**Conclusion:**

Our experiment verifies the hypothesis that ultrasound mediation of targeted microbubbles can enhance the transfection efficiency of shRNA-Livin in ovarian cancer cells.

## 1. Introduction

Ovarian cancer usually originates from the fallopian tube rather than the ovary. It refers to the epithelial cancer of the ovary or fallopian tube, as well as the histologically similar primary peritoneal cancer [1]. Ovarian cancer, known as the silent killer, is associated with 150,000 deaths in 2012, ranking eighth among the causes of cancer death in women [2]. Actually, more than 70% people who suffered from ovarian cancer are not diagnosed until the late stage due to the lack of effective screening tool, as well as vague symptoms, resulting in difficulty in cure [3]. It has been reported that the people with ovarian cancer were with 47.4% five-year survival [4]. The prevalence of ovarian cancer is positively correlated with age. Women under 40 years of age are more likely to have high probability of ovarian germ cell tumor rather than ovarian cancer. Ovarian cancer is common among women over the age of 40 especially in developed countries, which is the second most common malignancy after breast cancer among this group [5, 6].

At present, surgical resection of high-risk tissues is the most successful strategy in the treatment of ovarian cancer [7]. However, some reports indicated that surgery applied to advanced ovarian cancer frequently leads to serious postoperative complications, which directly affect survival [8, 9]. Therefore, it is of great significance to explore new treatments for ovarian cancer. The study found that abnormal expression of genes regulating cell apoptosis affects the occurrence and development of tumors [10, 11]. Inhibitor of apoptosis (IAP) family is antiapoptotic proteins with 70 amino acid baculovirus repeats [12] and involved in negative regulation of apoptosis [13, 14]. Livin, as a new important member of the IAP family proteins, is highly expressed in a variety of tumor cells, participates in the inhibition of cell apoptosis, and is closely related to the occurrence and development of tumors [15, 16]. The upregulation of Livin was found both in primary specimens from ovarian cancer patients and in ovarian cancer cell lines compared to normal controls. Overexpression of specific Livin transcripts promoted cell viability and migration, whereas Livin knockdown repressed these cellular processes. These effects of the Livin gene were also confirmed in a xenograft mouse model [15]. Ultrasound is the second most widely used imaging method worldwide. With the rapid development of ultrasound technology and material science, ultrasound contrast agent has expanded from the traditional diagnostic field to the therapeutic field. Ultrasound combined with appropriate delivery systems to target sites has been widely utilized [17]. Microbubbles with diameter 1–7 *μ*M are used as ultrasonic contrast agents, enhancing ultrasonic backscattered signals. The therapeutic drug coated by microbubbles is able to reach the target sites and then release [18, 19]. Streptavidin-biotin technology using microfluidic devices is effective in the synthesis of targeted lipid microbubbles, with precise size and high monodispersity than common lipid microbubbles prepared by DPPD and DSPE [20].

In the present study, lipid microbubbles bind to LHRHa targeting Livin, prepared by Shanghai Genechem Co., Ltd., which was applied to ovarian cancer cell OVCA-433. However, the low entrapment efficiency of the lipid microbubbles results in a lack of sufficient plasmid in the transfection process; hence, the microbubbles were mixed with an appropriate amount of plasmids for this research. This study aimed at exploring the inhibition effect of ultrasound-targeted microbubble destruction in Livin expression affecting apoptosis of ovarian cancer cell line OVCA-433 and providing reference for gene therapy in ovarian cancer.

## 2. Materials and Methods

### 2.1. OVCA-433 Cell Culture and Grouping

OVCA-433 cell line (Shanghai Suer Biotechnology Co., Ltd., China) was cultured in the DMEM (Tongpai Biotechnology Co., Ltd., Shanghai, China) containing 10% fetal calf serum (FBS), 100 U/ml penicillin, and 100 *μ*g/ml streptomycin at 37°C in an incubator with 5% CO_2_. When cells came to their logarithmic phase of growth, cell suspension (1 × 10^6^ cells/mL) was placed in the 24-well culture plate, and each well was added with 50 *μ*L. Afterward, OVCA-433 cells were assigned into blank, shRNA-Livin (transfection of shRNA-Livin alone), shRNA-Livin-NMB (shRNA-Livin plus nontargeted microbubbles), shRNA-Livin-TMB (shRNA-Livin plus LHRHa-conjugated microbubbles containing shRNA-Livin), shRNA-Livin + US (shRNA-Livin transfection followed by ultrasound destruction), shRNA-Livin-NMB + US (the mixture of shRNA-Livin and NMBs followed by ultrasound destruction), and shRNA-Livin-TMB + US (the mixture of shRNA-Livin and TMBs followed by ultrasound destruction) groups. The concentration of lipid microbubbles was 0.6 × 10^8^/mL. For ultrasound destruction, a 1 MHz piezoelectric ceramic transducer was immersed 2 mm above the cell suspension within the cell culture medium. Ultrasound pulses with an averaged intensity of 0.5 W/cm^2^ were applied to the medium for 8 s. Following exposure of the ultrasound pulses, the cells were seeded in a 24-well plate and incubated for 24 h.

### 2.2. Preparation of Nontargeted Lipid Microbubbles (NMBs)

1,2-Dipalmitoyl-sn-glycero-3-phosphocholine (DPPC) and 1,2-distearoyl-sn-glycero-3-phosphoethanolamine (DSPE) were dissolved in 50 *μ*l glycerol at a ratio of 10 : 1 (5 mg : 0.5 mg). After adding with 450 *μ*l phosphate-buffered saline (PBS), the mixture was placed in a 40°C water bath for 30 min and then refilled with perfluorobutane gas for shaking 45 s. The generated NMBs were washed with 0.5 ml PBS and stored at −20°C.

### 2.3. Preparation of Targeted Lipid Microbubbles (TMBs)

DPPC and DSPE-PEG2000-biotin were dissolved in 50 *μ*l glycerol at a ratio of 10 : 1 to biotinylate lipid microbubbles. Next, 1 *µ*l biotinylated lipid microbubbles were adjusted into 1 × 10^8^/ml, and then 100 *μ*l microbubbles were diluted into 1 ml in PBS. After centrifugation at 1000 rpm for 3 min, the supernatant was added with 60 *µ*g FITC-labeled streptavidin and underwent low-speed shake (180 cycles/min) for 20 min. After centrifugation at 1000 rpm for 3 min again, the supernatant was collected as avidinylating lipid microbubbles. The avidinylated microbubbles were conjugated with 50 biotinylated LHRHa peptides. The mixture of 10 *µ*l LHRHa-conjugated lipid microbubbles and 100 *µ*l poly-L-lysine was added with 1.6 *µ*g shRNA-Livin and incubated for 30 min. After PBS washing and centrifugation, TMBs were purified and stored at −20°C.

### 2.4. Cell Survival Assays

OVCA-433 cell survival was evaluated using MTT assays. In brief, cell suspension (logarithmic phase) was set as a concentration of 1 × 10^6^ cell/ml, detached by trypsase, supplemented with DMEM, and then placed into the 96-well culture plate with 150 *μ*L in each well. Three replications were prepared for each group. After 24 h incubation in the 5% CO_2_ incubator at 37°C, each well was added with 10 *μ*l MTT (5 mg/ml) and cultured for another 4 h. With the supernatant removed, each well was added with 100 *μ*l DMSO and shook by an oscillator at low speed for 10 min. The OD value was measured at the wavelength of 492 nm detected, and cell survival rate was calculated.

### 2.5. Cell Apoptosis Assays

In brief, the cells were harvested with trypsin (0.25%), washed twice with PBS, and adjusted into 1 × 10^6^ cell/ml. OVCA-433 cell apoptosis was determined by annexin V/PI double staining in strict accordance with the instructions of the kit (Shanghai Kanglang Biotechnology Co., Ltd., China). Apoptotic index = [(early apoptotic cells + late apoptotic cells)/total cells] × 100%.

### 2.6. RNA Extraction and Real-Time Quantitative PCR (RT-qPCR)

Total RNA of OVCA-433 cells in each group was extracted by the TRIzol method, and cDNA was generated by using a PrimeScript RT Reagent Kit (Takara, Dalian, China). RT-qPCR was performed with the SYBR^®^ Premix Ex Taq^™^ II (Tli RNaseH Plus) kit (Takara) using a ABI PRISM^®^ 7500 System (Applied Biosystems, Foster City, CA, USA). Primer synthesis was completed by Synbio Technologies (Suzhou, Jiangsu, China). Primer sequences for Livin were as follows: upstream: 5′-GGTCATTACTGGAGTCTTG-3′ and downstream: 5′-CACTTTAACAATAGGCGAGT-3′; for caspase-3: upstream: 5′-CTGATCCACAGGAGATATTA-3′ and downstream: 5′-CAATAATGCTGTAAAACCTT-3′; for caspase-8: upstream: 5′-GGTGGCATGTGCGGATGG-3′ and downstream: 5′-TTCAACGGTGAGGTCACG-3′; for GAPDH: upstream: 5′-TTCGACAGTCAGCCGCATCTT-3′ and downstream: 5′-CCCAATACGACCAAATCCGTT-3′. The reaction system adopted 40 cycles, which included 2 min of predenaturing at 94°C, 30 s of annealing at 94°C, 30 s of extension at 52°C, and 1 min of extension at 72°C. The relative expressions of Livin, caspase-3, and caspase-8 mRNA were calculated using 2^−ΔΔCt^ methods with GAPDH mRNA expression as the internal reference.

### 2.7. Western Blotting

The total protein of OVCA-433 cells in each group was extracted by RIPA lysis buffer and quantified by the BCA method. The protein was separated by SDS-PAGE and wet-transferred onto the PVDF membrane. The PVDF membrane was sealed using 5% skimmed milk powder for 1 h, probed with anti-Livin antibody (ab236500, Abcam, Cambridge, UK), anti-caspase-3 antibody (ab32351, Abcam), anti-caspase-8 antibody (ab32397, Abcam), and anti-GAPDH antibody (ab8245, Abcam) overnight at 4°C, and then reprobed with secondary antibody IgG conjugated with HRP. Western blots were rinsed with TBST buffer for 3 times and reacted with ECL solutions.

### 2.8. Statistical Analysis

SPSS 20.0 software was used for data analysis. Data were expressed as mean ± standard deviation, and *t*-test was performed for two-group comparison, one-way ANOVA for multiple-group comparison, and two-way ANOVA for comparison at different time points. *P* < 0.05 was considered as statistically significant.

## 3. Results

### 3.1. UTMD Enhanced the Inhibition of shRNA-Livin on OVCA-433 Cell Survival

The cell survival rates of each group were calculated at indicated culture time points, 24, 48, and 72 h (Table 1). After culture for 24 h, the cell survival rates in the shRNA-Livin-TMB, shRNA-Livin + US, shRNA-Livin-NMB + US, and shRNA-Livin-TMB + US groups were lower than those in the blank, shRNA-Livin, and shRNA-Livin-NMB groups (*P* < 0.05); after culture for 48 h, the cell survival rate in the shRNA-Livin-NMB group was lower than that in the blank and shRNA-Livin groups (*P* < 0.05); after culture for 72 h, the cell survival rate in the shRNA-Livin group was lower than that in the blank group (*P* < 0.05), suggesting that UTMD enhanced the efficacy of shRNA-Livin to reduce OVCA-433 cell survival. The shRNA-Livin-TMB + US group exhibited the lowest cell survival rate among the 7 groups (*P* < 0.05), indicating that UTMD enhanced the inhibition of shRNA-Livin on OVCA-433 cell survival.

### 3.2. UTMD Enhanced the Promotion of shRNA-Livin on OVCA-433 Cell Apoptosis

**Figure 1:**
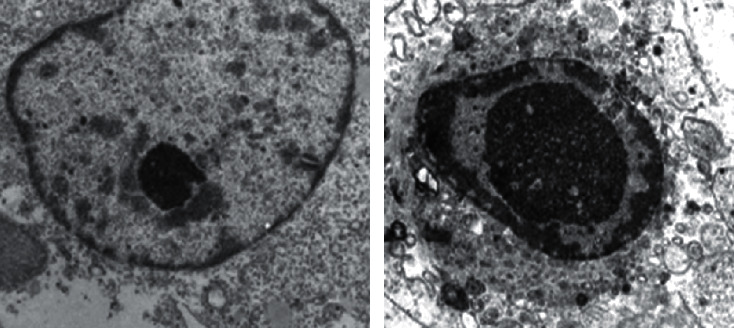
Cell ultrastructure (×2000) of the blank group (a) and shRNA-Livin-TMB + US group (b).

After culture for 48 h, it was found that the nucleus of OVCA-433 cells in the blank group was larger, with complete organelles seen in the cytoplasm under the transmission electron microscope. Early apoptosis represented by changes in the nucleolus and cytoplasm gathering under the nuclear membrane was noted in OVCA-433 cells in the shRNA-Livin-TMB + US group. Results of flow cytometry using annexin V/PI double staining revealed the apoptosis rates of the blank, shRNA-Livin, shRNA-Livin-NMB, shRNA-Livin-TMB, shRNA-Livin + US, shRNA-Livin-NMB + US, and shRNA-Livin-TMB + US groups to be 3.41 ± 0.37%, 6.15 ± 0.54%, 6.29 ± 0.81%, 6.91 ± 0.75%, 8.14 ± 0.93%, 12.95 ± 1.37%, and 29.51 ± 3.87%, respectively. Compared with the blank group, the other 6 groups showed remarkable increases in OVCA-433 cell apoptosis rate (*P* < 0.05), and the shRNA-Livin-TMB + US group indicated much higher cell apoptosis rate compared to the shRNA-Livin, shRNA-Livin-NMB, shRNA-Livin-TMB, shRNA-Livin + US, and shRNA-Livin-NMB + US groups (*P* < 0.01, Figure 1). These data suggested that UTMD enhanced the promotion of shRNA-Livin on OVCA-433 cell apoptosis.

### 3.3. UTMD Enhanced the Increase of shRNA-Livin on Caspase-3 and Caspase-8 mRNA

As listed in Table 2, after 48 h of culture, the relative expressions of Livin mRNA were lower, but the relative expressions of caspase-3 mRNA and caspase-8 mRNA were higher in shRNA-Livin, shRNA-Livin-NMB, shRNA-Livin-TMB, shRNA-Livin + US, shRNA-Livin-NMB + US, and shRNA-Livin-TMB + US groups than those in the blank group (*P* < 0.05). The shRNA-Livin-TMB + US group indicated a lower expression of Livin mRNA and higher expressions of caspase-3 mRNA and caspase-8 mRNA compared to the shRNA-Livin, shRNA-Livin-NMB, shRNA-Livin-TMB, shRNA-Livin + US, and shRNA-Livin-NMB + US groups (*P* < 0.01).

### 3.4. UTMD Enhanced the Increase of shRNA-Livin on Caspase-3 and Caspase-8 Proteins

**Figure 2:**
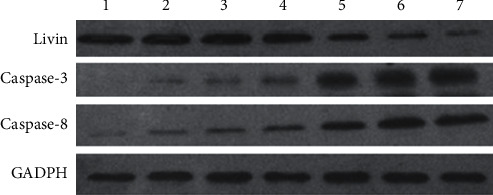
Western blots of Livin, caspase-3, and caspase-8 in OVCA-433 cells. No. 1, blank group; no. 2, shRNA-Livin group; no. 3, shRNA-Livin-NMB group; no. 4, shRNA-Livin-TMB group; no. 5, shRNA-Livin + US group; no. 6, shRNA-Livin-NMB + US group; no. 7, shRNA-Livin-TMB + US group.

After 48 h of culture, the relative expressions of Livin protein were lower, but the relative expressions of caspase-3 and caspase-8 proteins were higher in shRNA-Livin, shRNA-Livin-NMB, shRNA-Livin-TMB, shRNA-Livin + US, shRNA-Livin-NMB + US, and shRNA-Livin-TMB + US groups than those in the blank group (*P* < 0.05). The shRNA-Livin-TMB + US group indicated a lower expression of Livin protein and higher expressions of caspase-3 and caspase-8 proteins compared with the shRNA-Livin, shRNA-Livin-NMB, shRNA-Livin-TMB, shRNA-Livin + US, and shRNA-Livin-NMB + US groups (*P* < 0.01, Figure 2 and Table 3).

## 4. Discussion

Ovarian cancer is the most lethal malignant tumor in gynecological tumors. The incidence rate of ovarian cancer is lower than endometrial cancer [21]. In 2012, about 145,000 deaths of ovarian cancer worldwide [2] were reported. Ovarian cancer is characterized by vague symptoms and low 5-year survival rate. It was reported that 70% women died of this disease after surgery or chemotherapy [22]. The low survival rate is mainly associated with postoperative complications [23] and cancer drug resistance [24]. Ovarian cancer cell line OVCA-433 ranks first among drug-resistant cell lines, and its drug resistance index is higher than 300 presented in 75% of drugs [25].

Excessive cell proliferation and slow apoptosis caused by abnormal cell cycle regulation play an important role in the occurrence and development of tumors. Cell apoptosis and caspase activation pathway mediate cell death, and this process is performed by various proteins [26]. Livin is a member of the IAP family. It is a functional inhibitor of apoptosis and a structure-related protein, acting as an endogenous inhibitor of apoptosis. Reducing the activity of the IAP family to induce tumor cell apoptosis has become a research hotspot in the field of gene therapy in tumor and cancer [27]. Hendruschk et al. mentioned that low expression of antiapoptotic survivin through RNA interference technology induced glioma cell apoptosis and damaged cell proliferation [28]. A study reported by Lv et al. revealed that inhibition of Livin using RNA interference technology contributed to the apoptosis of leukemia cell line K562 [29]. However, RNA interference technology has the problems of low transfection rate in the vector gene and poor specific targeting, which seriously affects the effect of gene therapy in tumor. Therefore, exploring stable transfection methods with high transfection efficiency, selectivity, and specificity is the key to improve gene therapy.

In recent years, with the development of treatment technology and the emergence of targeted ultrasound contrast agents developing specificity in the target area, microbubbles have become an interesting carrier in gene and drug delivery for tumor and cancer treatment [30, 31]. In this study, 7 cell groups with different treatments were involved in this experiment. This study focused on the effects of the group receiving lipid microbubbles targeting Livin gene as the ultrasound contrast agent in the proliferation and apoptosis of ovarian cancer cell line OVCA-433. The results showed that, after cell culture at 24 h, 48 h, and 72 h, the cell group receiving ultrasound-targeted microbubble destruction revealed a significant lower cell survival rate than in other 6 cell groups. In addition, after 48 h of cell culture, the cell apoptosis rate in this group was significantly higher compared to other 6 cell groups. It was suggested that ultrasound-targeted microbubble destruction contributed to effective inhibition of Livin in ovarian cancer cells, resulting in accelerating cell apoptosis and reducing cell survival rate. These findings were similar to another study, indicating that overexpression of sirtuin-3 mediated by targeted microbubble destruction was helpful to inhibit the progression of ovarian cancer [32]. The research presented by Chen et al. pointed out that ultrasound microbubble destruction targeting survivin induced apoptosis of HeLa cells in cervical cancer and led to inhibit the progression of cervical cancer [33].

At present, it is considered that apoptosis is mainly mediated by two signal pathways, including the death receptor signal pathway [34] and mitochondria-independent signal pathway [35]. Caspase-3 is identified as a key mediator of apoptosis in neuronal cells in the death receptor pathway and mitochondria-independent pathway [36]. Caspase-8 is the key to the regulation and activation of cell death mediated by the death receptor signal pathway [37]. In general, the antiapoptotic activity of Livin is mediated by inhibiting caspase. Western blot analysis in this study manifested that, compared to the remaining groups, remarkable low expression of Livin mRNA and Livin protein was found in the cell group treated with ultrasound-targeted microbubble destruction; besides, this group indicated significant overexpression of caspase-3 and caspase-8 mRNA as well as protein. The findings revealed that the antiapoptotic activity of Livin was negatively correlated with the expression of caspase-3 and caspase-8. Ultrasound-targeted microbubble destruction accelerated the apoptosis of ovarian cancer OVCA-433 cells by upregulating the expression of caspase-3 and caspase-8. In the research of prostatic cancer, Gu et al. suggested that excessive expression of Livin was found in prostatic cancer tissue, and its expression was negatively correlated with caspase-3 expression [38]. A similar study reported by Jin et al. revealed that, after irradiation, significant higher activity of caspase-3 and caspase-8, and low expression of Ki67, survivin and Livin were found in the lung carcinoma xenografts [39]. All these findings demonstrated that Livin was negatively correlated with caspase-3 and caspase-8.

In summary, ultrasound-targeted microbubble destruction targeting Livin has been proven as an effective approach to inhibit proliferation and induce apoptosis of ovarian cancer cells. The antiapoptotic activity of Livin was associated with expressions of caspase-3 and caspase-8. However, due to the low entrapment efficiency of targeted microbubbles, recombinant plasmid was added into the cultured ovarian cancer cell. For further investigation, addition of different concentrations of microbubbles and exposure to different intensities and times of ultrasound, such as 0.5, 1.0, and 1.5 W/cm^2^ and 8, 30, and 50 s, in more than single ovarian cancer cell line were required for the application of UTMD in gene therapy of ovarian cancer.

## Figures and Tables

**Table 1:** Cell survival rate of each group at different culture time points.

Group	24 h (%)	48 h (%)	72 h (%)
Blank	99.15 ± 0.39^②^	98.39 ± 0.55^②^	94.07 ± 0.43^②^
shRNA-Livin	97.39 ± 0.62^②^	94.51 ± 0.90^②^	82.16 ± 1.39^①②^
shRNA-Livin-NMB	96.42 ± 0.80^②^	90.25 ± 0.61^①②^	75.43 ± 0.82^①②^
shRNA-Livin-TMB	94.26 ± 1.42^①②^	86.09 ± 0.73^①②^	70.27 ± 1.04^①②^
shRNA-Livin + US	79.13 ± 1.85^①②^	71.64 ± 1.30^①②^	47.81 ± 2.71^①②^
shRNA-Livin-NMB + US	70.51 ± 1.59^①②^	55.17 ± 1.88^①②^	39.25 ± 1.18^①②^
shRNA-Livin-TMB + US	58.37 ± 2.15^①^	44.32 ± 1.45^①^	24.49 ± 1.06^①^

^①^ *P* < 0.05 compared to the blank group; ^②^*P* < 0.05 compared to the shRNA-Livin-TMB + US group.

**Table 2:** The mRNA expression levels of Livin, caspase-3, and caspase-8 in each group.

Group	Livin	Caspase-3	Caspase-8
Blank	4.95 ± 0.35^②^	0.95 ± 0.19^②^	1.50 ± 0.15^②^
shRNA-Livin	4.60 ± 0.31^②^	1.13 ± 0.15^②^	1.68 ± 0.23^②^
shRNA-Livin-NMB	4.43 ± 0.23^②^	1.30 ± 0.26^②^	1.83 ± 0.21^②^
shRNA-Livin-TMB	3.35 ± 0.46^①②^	1.52 ± 0.21^①②^	2.25 ± 0.27^①②^
shRNA-Livin + US	2.49 ± 0.15^①②^	2.76 ± 0.18^①②^	3.62 ± 0.35^①②^
shRNA-Livin-NMB + US	2.06 ± 0.13^①②^	3.15 ± 0.47^①②^	3.95 ± 0.31^①②^
shRNA-Livin-TMB + US	1.33 ± 0.08^①^	4.38 ± 0.45^①^	5.08 ± 0.63^①^

^①^ *P* < 0.05 compared to the blank group; ^②^*P* < 0.05 compared to the shRNA-Livin-TMB + US group.

**Table 3:** The protein expression levels of Livin, caspase-3, and caspase-8 in each group.

Group	Livin	Caspase-3	Caspase-8
Blank	2.71 ± 0.35^②^	0.12 ± 0.02^②^	0.35 ± 0.08^②^
shRNA-Livin	2.54 ± 0.31^②^	0.16 ± 0.04^②^	0.49 ± 0.15^②^
shRNA-Livin-NMB	2.49 ± 0.28^②^	0.18 ± 0.04^②^	0.55 ± 0.11^②^
shRNA-Livin-TMB	2.30 ± 0.33^①②^	0.22 ± 0.06^①②^	0.71 ± 0.18^①②^
shRNA-Livin + US	1.68 ± 0.21^①②^	0.96 ± 0.14^①②^	1.43 ± 0.20^①②^
shRNA-Livin-NMB + US	1.35 ± 0.18^①②^	1.21 ± 0.29^①②^	1.85 ± 0.27^①②^
shRNA-Livin-TMB + US	0.82 ± 0.13^①^	1.94 ± 0.37^①^	2.50 ± 0.33^①^

^①^ *P* < 0.05 compared to the blank group; ^②^*P* < 0.05 compared to the shRNA-Livin-TMB + US group.

## Data Availability

The data used to support the findings of this study are included within the article.
